# Colchicine inhibits vascular calcification by suppressing inflammasome activation through the enhancement of the Sirt2-PP2Ac signaling pathway

**DOI:** 10.1016/j.jbc.2025.110381

**Published:** 2025-06-14

**Authors:** Shu Yang, Heming Huang, Kewei Jiang, Ying Peng, Zhen Liang, Xinyu Gong, Lixing Li, Yanchun Li, Buchun Zhang, Yuanli Chen, Xiaoxiao Yang

**Affiliations:** 1 Department of Geriatrics, The First Affiliated Hospital of Southern University of Science and Technology, Shenzhen, Guangdong, China; 2 Anhui Provincial International Science and Technology Cooperation Base for Major Metabolic Diseases and Nutritional Interventions, College of Food and Biological Engineering, Hefei University of Technology, Hefei, Anhui, China; 3 Department of Cardiology, the First Affiliated Hospital of USTC, Division of Life Sciences and Medicine, University of Science and Technology of China, Hefei, Anhui, China

**Keywords:** Sirt2, colchicine, vascular calcification, NLRP3, acetylation, phosphorylation

## Abstract

Colchicine (Col) is a traditional herbal medicine derived from the plant *Colchicum autumnale*. With the property of anti-inflammation, Col has demonstrated certain therapeutic effects in cardiovascular diseases. Vascular calcification is positively related to the morbidity and mortality of cardiovascular diseases. However, the specific cardiovascular conditions for which Col is effective remain unclear, particularly its impact on vascular calcification. In this study, we used high phosphate to induce calcium deposition in vascular smooth muscle cells and Vitamin D_3_ plus nicotine or 5/6 nephrectomy along with high phosphate diet to construct vascular calcification mouse models. Our results showed that Col reduced calcium accumulation *in vitro*, and vascular calcification both in *ex vivo* and *in vivo* models, which was evidenced by the Alizarin red S staining and calcium content determination. *In vitro* results showed that Col-inhibited vascular calcification is contributed to the reduction of NLR family pyrin domain-containing 3 inflammasome activation through enhanced phosphorylation at Ser 5. In addition, we indicated that phosphorylation of NLR family pyrin domain-containing 3 is regulated by the activity of protein phosphatase 2Ac. Furthermore, we identified Sirt2 as a master regulator of protein phosphatase 2Ac activation through regulating its acetylation at Lys 136. More importantly, we demonstrated that Col-inhibited vascular calcification is dependent on Sirt2 expression by using the Sirt2 knockout mice. Collectively, we demonstrate that Col protects against vascular calcification. Our study provides novel insight into the clinical application of Col. We also suggest that Sirt2 is a novel target for vascular calcification treatment and that Col may act as an activator of Sirt2, which could be beneficial in other diseases.

Cardiovascular diseases (CVDs) are a type of chronic disease with complex pathogenesis. Vascular calcification is a significant late-stage phenotype, an important risk factor of CVDs (1). Vascular calcification is characterized as mineral deposition with the form of calcium–phosphate complexes. The severity of vascular calcification reflects plaque burden and independently predicts morbidity and mortality of cardiovascular diseases (2). Vascular smooth muscle cells (VSMCs) dysfunction is a major cause of cardiovascular diseases (3, 4). Phenotypic plasticity is a main feature of VSMCs. Vascular calcification is initiated by the de-differentiation of VSMCs, with the features of proliferation, migration, senescence, osteogenesis, and mineralization, which was caused by vascular injury and environmental stimulus, such as inflammatory cytokines, adhesion molecules, advanced glycation end products, and high phosphate (4, 5). In addition to osteogenic differentiation, it has been reported that VSMCs can develop to fibroblasts, macrophage-like cell, and even foam cell, which also promote the development of vascular calcification (6, 7, 8). Runt-related transcription factor-2 (RUNX2), the master osteogenic transcription factor, as well as bone matrix proteins and osteogenic regulators, such as osteopontin (OPN) and bone morphogenetic protein 2 (BMP2), were enhanced in response to phosphate stress, which lead to the osteogenic differentiation and calcification of VSMCs (9, 10, 11, 12, 13). However, it is still lacking the effective therapeutic strategies for vascular calcification.

Colchicine (Col) is originally extracted from the autumn crocus (*Colchicum autumnale*) and has been found over centuries. It was used as an anti-inflammatory drug to treat gout and familial Mediterranean fever (14). Due to function of inhibition inflammasome assembly, impairment of neutrophil chemotaxis, adhesion, and recruitment, and disruption the interaction of neutrophil-platelet, it plays important role in cardiovascular diseases, including pericarditis, acute and chronic coronary syndromes, fibrillation, and heart failure (14, 15, 16, 17, 18). It is well-established and recommended by guidelines for pericarditis treatment. The research on the exact role of Col on fibrillation and heart failure is still ongoing and need to be well-defined (17, 19). Both clinical trials and basic researches indicate that Col performs beneficial roles in coronary diseases. However, studies have shown that not all patients benefit from Col (20). Recently, a randomized, double-blind clinical trial demonstrated that low-dose Col (0.5 mg/day for 12 months) promotes coronary plaque stabilization in patients with acute coronary syndrome and supported the potential role of Col in secondary prevention strategies (21). Due the complex mechanisms and pathogenesis, the specific cardiovascular conditions for which Col is effective remain unclear, particularly its impact on vascular calcification in advanced atherosclerosis, which has not been previously reported. This study aims to elucidate the effects of Col on vascular calcification, thereby enhancing its precise application in clinical practice.

In this current study, we used high phosphate to induced calcium deposition in VSMC, and Vitamin D_3_ (VD_3_) plus nicotine or 5/6 nephrectomy combined with high phosphate diet to construct vascular calcification mouse model for elucidation of the role of Col on calcification, as well as the involved mechanisms.

## Results

### Col attenuates calcification

**Figure 1:**
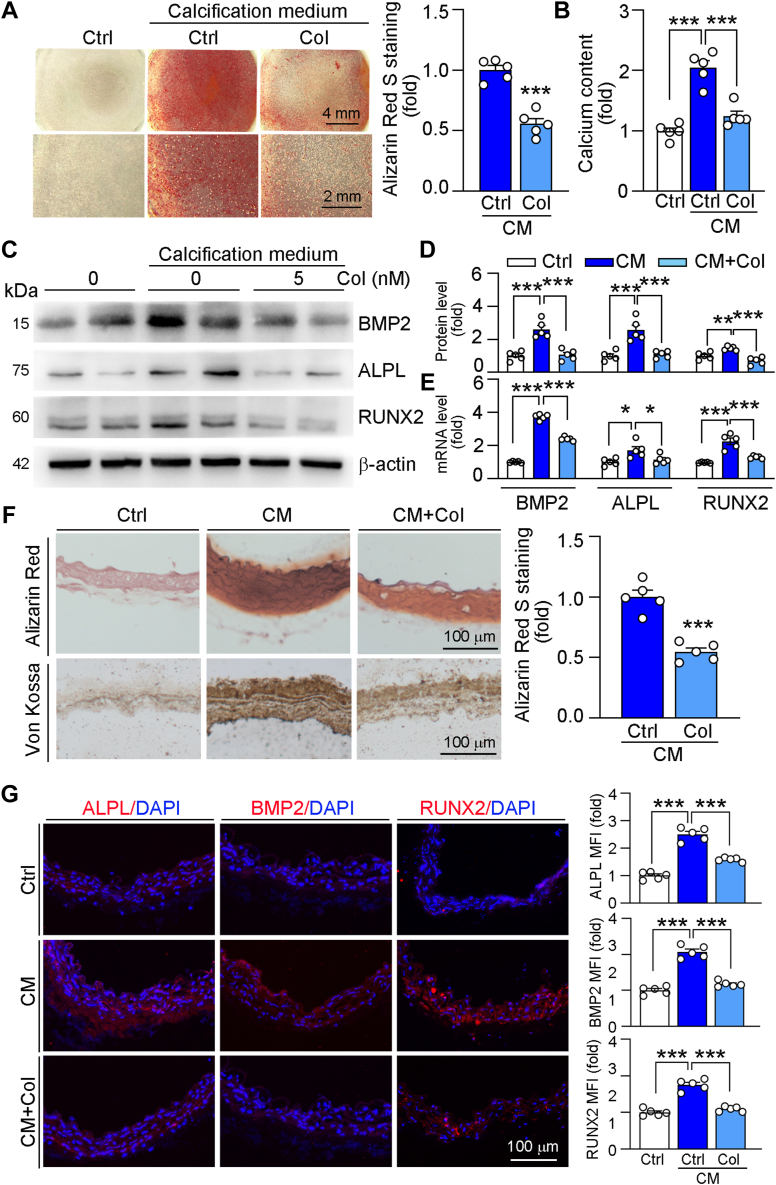
**Col inhibits calcification both *in vitro* and *ex vivo***. *A*–*E*, human VSMCs were cultured in 2% FBS and 1% P/S medium (control medium) or high phosphate medium (CM, control medium containing 3 mM phosphate) or CM containing col (5 nM) for 4 days. *A*, Alizarin red S staining was used to determine calcification, followed by quantitative analysis (n = 5). *B*, calcium content was quantified using the indicated assay kit (n = 5). *C* and *D*, ALPL, BMP2, and RUNX2 protein expression was determined by Western blot with quantification of band density (n = 5). *E*, ALPL, BMP2, and RUNX2 mRNA levels were determined by qRT-PCR (n = 5). *F* and *G*, thoracic aortas were collected from C57BL/6J mice and cut into 5 mm long aortic rings, then cultured in control medium or CM or CM containing col (5 nM) for 14 days. Alizarin red S staining was used to determine calcification, followed by quantitative analysis (*F*, n = 5). Protein expression of ALPL, BMP2, and RUNX2 was determined by immunofluorescent staining with quantification of MFI (*G*, n = 5). For all *panels*, data are presented as mean ± SD. ∗*p* < 0.05, ∗∗*p* < 0.01, and ∗∗∗*p* < 0.001 by one-way ANOVA with Bonferroni correction test. ALPL, alkaline phosphatase; BMP2, bone morphogenetic protein 2; CM, calcification medium; MFI, mean fluorescence intensity; RUNX2, Runt-related transcription factor-2; VSMCs, vascular smooth muscle cells.

Before the experiment, we determined the cellular toxicity of Col and found that low concentration of Col had little effects on cell viability (Supplementary material online, Fig. S1*A*). Then, we used high phosphate medium to induce VSMCs calcification and treated cells with Col. The results of Alizarin red S staining and calcium content showed that Col largely reduced calcification (Fig. 1, *A* and *B*). Consistently, alkaline phosphatase (ALPL), BMP2, and RUNX2, the osteogenic markers were significantly upregulated in the high phosphate–treated VSMCs, while reduced by Col treatment (Fig. 1, *C* and *D*). Moreover, we also found that Col reduced the high phosphate–induced mRNA levels of ALPL, BMP2, and RUNX2 in VSMCs (Fig. 1*E*). Furthermore, we conducted *ex vivo* study and found that Col reduced calcification in aortic ring of wildtype mice (Fig. 1*F*). Additionally, the results of immunofluorescent staining indicated that osteogenic markers were also inhibited by the treatment of Col (Fig. 1*G*).

**Figure 2:**
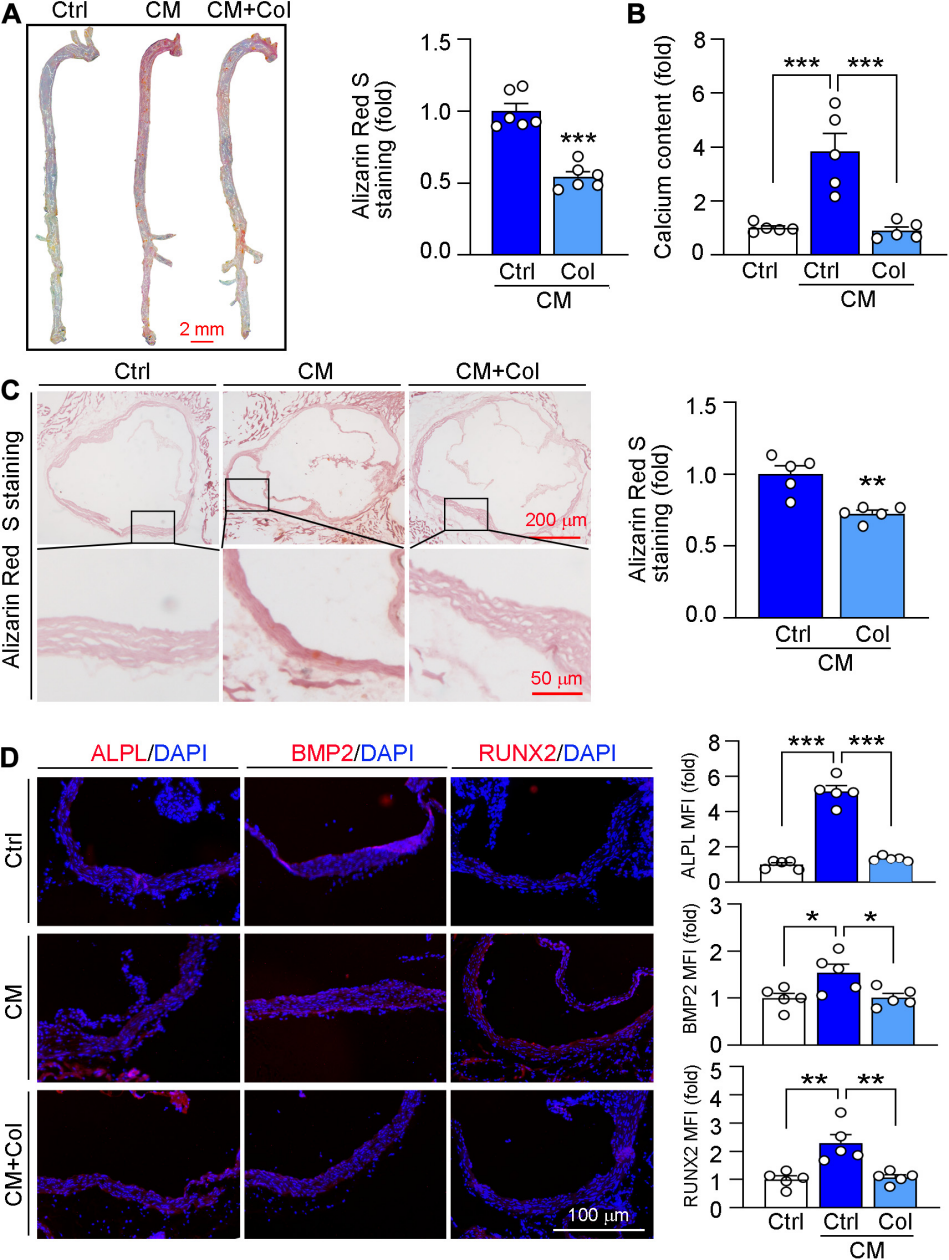
**Col inhibits calcification *in vivo***. C57BL/6J mice were injected with VD_3_ and intragastrically administered with nicotine to construct vascular calcification mouse model and fed normal food or food containing Col for 4 weeks. At the end of experiment, mice were euthanized for collection of aorta samples. *A* and *C*, vascular calcification in whole aorta (*A*, n = 6) and aortic root (*C*, n = 5) was determined by Alizarin Red S staining, followed by quantitative analysis. *B* and *D*, calcium content was determined by indicated assay kit (*B*, n = 5). Protein expression of ALPL, BMP2, and RUNX2 was determined by immunofluorescent staining with quantification of MFI (*D*, n = 5). For all panels, data are presented as mean ± SD. ∗*p* < 0.05, ∗∗*p* < 0.01, ∗∗∗*p* < 0.001 by one-way ANOVA with Bonferroni correction test. ALPL, alkaline phosphatase; BMP2, bone morphogenetic protein 2; Col, colchicine; MFI, mean fluorescence intensity; RUNX2, Runt-related transcription factor-2; VD_3_, vitamin D_3_.

VD_3_ plus nicotine was used for construction of vascular calcification mouse model and exploration of the role of Col on calcification *in vivo*. Our results showed that Col treatment largely reduced calcification in mouse aorta (Fig. 2*A*). Consistently, calcium content was also reduced by Col (Fig. 2*B*). Then, we showed that calcification in aortic root was also inhibited by Col (Fig. 2*C*). In line with the *in vitro* and *ex vivo* results, the results of Figure 2*D* showed that ALPL, BMP2, and RUNX2 were reduced by Col *in vivo*. Therefore, our data indicated that Col could reduce vascular calcification both *in vitro* and *in vivo*.

### Col attenuates calcification by reducing NLR family pyrin domain-containing 3 activation

**Figure 3:**
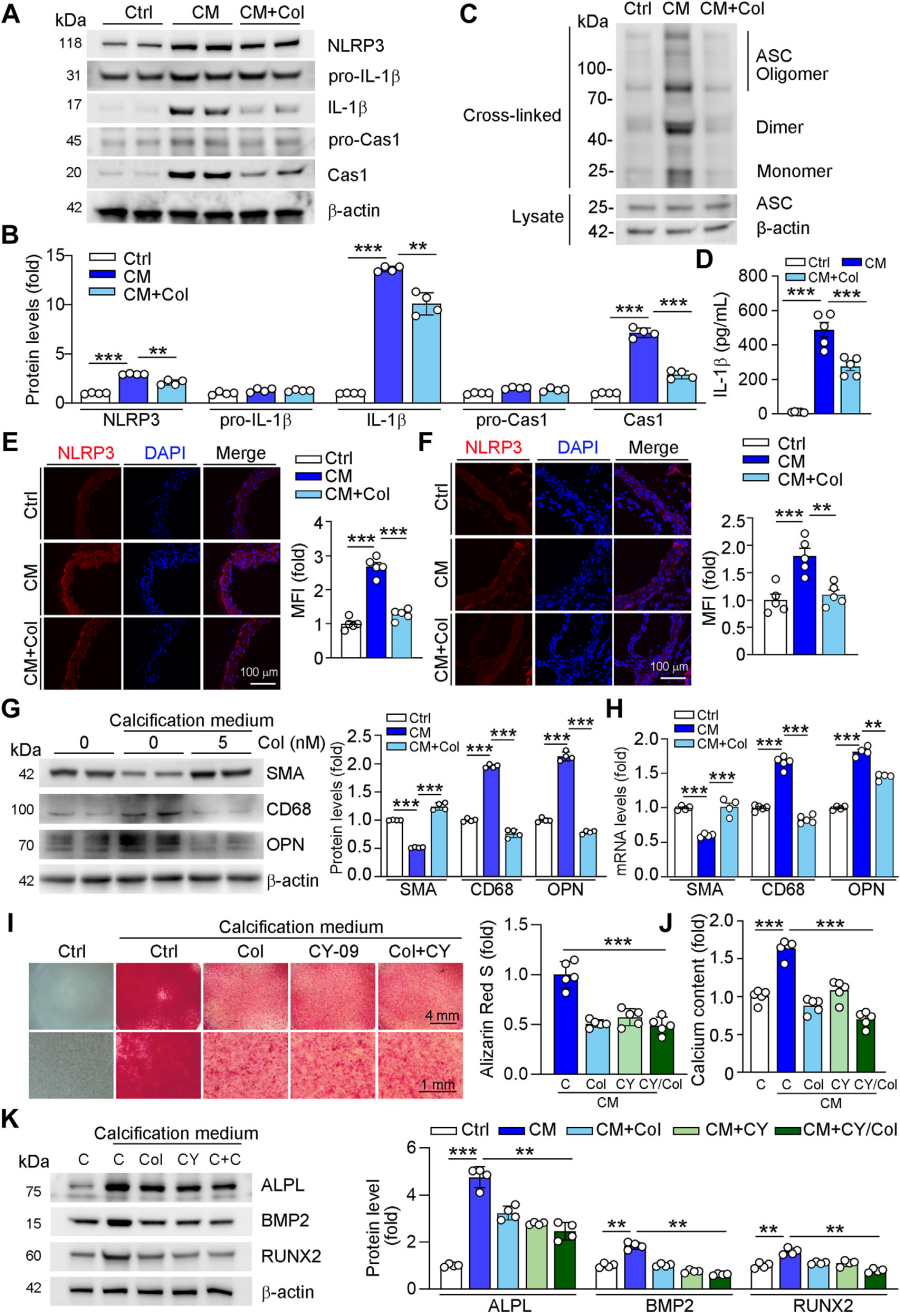
**Col inhibits NLRP3 activation**. *A*–*D*, human VSMCs were cultured in 2% FBS and 1% P/S medium or high phosphate medium (CM) or CM containing col (5 nM) for 4 days. NLRP3, pro-IL-1β, IL-1β, pro-Caspase1, and Caspase1 protein expression was determined by Western blot with quantification of band density, n = 4 (*A* and *B*). ASC oligomerization in cross-linked cytosolic pellets and the whole cell lysate was determined by Western blot (*C*). Cell medium was used for determination IL-1β levels, n = 5 (*D*). *E* and *F*, immunofluorescent staining was used to determine NLRP3 expression with quantification of MFI on sections of aorta collected from Figure 1*F* (*E*, n = 5) and 2 (*F*, n = 5). *G*–*K*, human VSMCs were cultured in 2% FBS and 1% P/S medium or high phosphate medium (CM) or CM containing col (5 nM) in the present or absence of CY-09 (10 μM) for 4 days. CD68, SMA, and OPN protein or mRNA expression was determined by Western blot (*G*, n = 4) or qRT-PCR (*H*, n = 4). Alizarin Red S staining was used to determine calcification followed by quantitative analysis (*I*, n = 5), and calcium content was quantified using the indicated assay kit (*J*, n = 5). ALPL, BMP2, and RUNX2 protein expressions were determined by Western blot with quantification of band density (*K*, n = 4). For all *panels*, data are presented as mean ± SD. ∗∗*p* < 0.01 and ∗∗∗*p* < 0.001 by one-way ANOVA with Bonferroni correction test. ALPL, alkaline phosphatase; ASC, apoptosis-associated speck-like protein with a caspase recruitment domain; BMP2, bone morphogenetic protein 2; CM, calcification medium; Col, colchicine; MFI, mean fluorescence intensity; NLRP3, NLR family pyrin domain-containing 3; OPN, osteopontin; RUNX2, Runt-related transcription factor-2; VSMCs, vascular smooth muscle cells.

It has been demonstrated that NLR family pyrin domain-containing 3 (NLRP3) can be inactivated by Col. However, it is still unclear whether NLRP3 is involved in Col-regulated calcification. In this study, we found that NLRP3 was activated by high phosphate, while reduced by Col treatment. Associated with NLRP3 inactivation, Col also reduced the high phosphate-enhanced mature form of interleukin-1β (IL-1β), caspase-1, as well as apoptosis-associated speck-like protein with a caspase recruitment domain (ASC) oligomerization but had little role in pro-IL-1β or pro-caspase-1 (Fig. 3, *A*–*C*). In addition, Col also reduced IL-1β secretion into cell culture medium (Fig. 3*D*). Consistent with the *in vitro* results, we found that NLRP3 was also reduced by Col in aortic ring (Fig. 3, *E* and *F*). NLRP3 plays dominant role in inflammation and macrophages activation. Previous study has shown that macrophage-like VSMCs can upregulate inflammation-associated genes (22). To explore the role of Col on phenotype transformation of VSMCs to macrophage-like VSMCs. We detected CD68, a marker of macrophage-like VSMCs, and found high phosphate–induced CD68 levels, which was inhibited by Col treatment (Figs. 3, *G* and *H*, and S2). Furthermore, high phosphate reduced the contractile phenotype marker, SMA, while enhanced the osteogenic phenotype marker OPN. However, Col treatment reversed the high phosphate–regulated SMA and OPN levels (Figs. 3, *G* and *H*, and S2). To determine the role of NLRP3 on macrophage-like VSMCs differentiation, we treated VSMCs with CY-09, an inhibitor of NLRP3, and found that CY-09 or cotreatment of CY-09 and Col largely reduced high phosphate–induced calcification and calcium content (Fig. 3, *I* and *J*), indicating the crucial role of NLRP3 in VSMCs phenotype differentiation. Furthermore, we found that osteogenic markers were reduced by treatment of CY-09 or cotreatment of CY-09 and Col (Fig. 3*K*).

### Col regulates NLRP3 phosphorylation at Ser 5 through the protein phosphatase 2Ac

**Figure 4:**
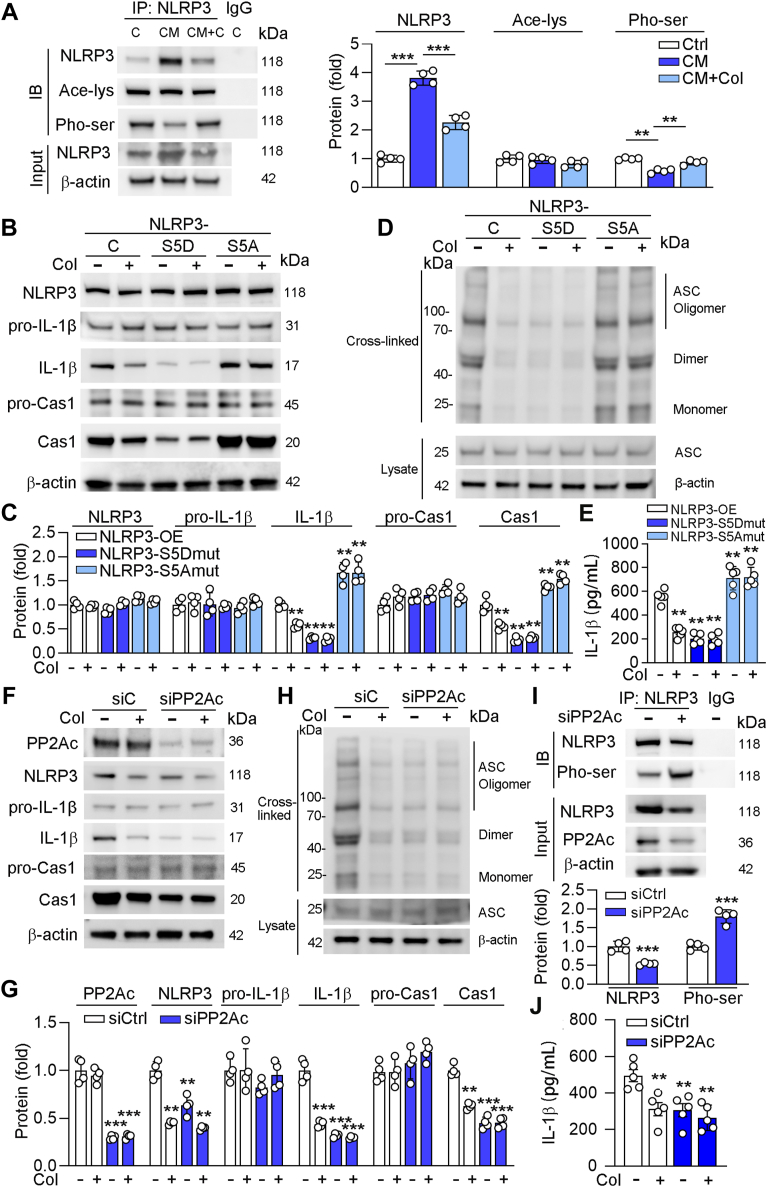
**Col enhances NLRP3 phosphorylation through PP2Ac**. *A*, human VSMCs were cultured in control medium or CM or CM containing Col for 4 days. *B–I*, human VSMCs were transfected with NLRP3, NLRP3-S5D, NLRP3-S5A, or siRNA for PP2Ac for 6h and transferred to complete medium for another 18 h, then cultured in control medium or CM or CM containing Col for another 4 days. For co-IP assay of NLRP3, cell lysates were immunoprecipitated with control IgG antibody or anti-NLRP3 antibody, followed by determination protein expression by Western blot using the indicated antibodies (*A*, and *I*, n = 4). For determination protein expression, cell lysates were used for conduction of Western blot (*B* and *F*, n = 4) with quantification of band density (*C* and *G*, n = 4). For determination ASC oligomerization, cell lysates were used for extracted cross-linked cytosolic pellets and determined by Western blot (*D* and *H*, n = 4). *J*, cell medium was collected for determination of IL-1β levels using the Elisa assay kit (*E* and *J*, n = 5). For all *panels*, data are presented as mean ± SD. ∗∗*p* < 0.01, ∗∗∗*p* < 0.001 by one-way ANOVA with Bonferroni correction test. ASC, apoptosis-associated speck-like protein with a caspase recruitment domain; CM, calcification medium; Col, colchicine; NLRP3, NLR family pyrin domain-containing 3; PP2Ac, protein phosphatase 2Ac; VSMCs, vascular smooth muscle cells.

We showed that NLRP3 activation is involved in Col-inhibited calcification. Previous studies have shown that the activation of NLRP3 can be regulated by acetylation and phosphorylation (23, 24). To explore whether acetylation or phosphorylation of NLRP3 is associated with Col-regulated calcification. We first determined the total acetylation and phosphorylation of NLRP3 and found that neither high phosphate nor Col had effects on NLRP3 acetylation. However, phosphorylation level of NLRP3 was reduced by high phosphate treatment but enhanced by Col (Fig. 4*A*). It was shown that phosphorylation at Ser 5 of NLRP3 (in the pyrin domain) inhibits inflammasome activation (23). Recently, some studies have shown that Bruton tyrosine kinase and PP2A can regulate NLRP3 activity through the phosphorylation at Ser 5 (25, 26). To determine the importance of Ser 5 in NLRP3 activation, we constructed adenovirus of NLRP3-S5D (the form of continuous phosphorylation) and NLRP3-S5A (the form of nonphosphorylation), and our results showed that phosphorylation level of NLRP3 was reduced by Col treatment in NLRP3-transfected cells but had little role in mutated NLRP3–transfected cells (Supplementary material online, Fig. S3). In addition, we found that the activation of IL-1β and caspase-1, as well as oligomerization of ASC was largely reduced in cells transfected with NLRP3-S5D, while enhanced by NLRP3-S5A. Moreover, Col had little inhibitory effects on IL-1β and caspase-1 in cells transfected with mutated NLRP3 (Fig. 4, *B*–*D*). Moreover, the secretion levels of IL-1β in medium was consistent with the mature form of IL-1β (Fig. 4*E*). Therefore, we indicated that Col-reduced NLRP3 activation is related to its phosphorylation at Ser 5.

Previous study has shown that Col reduces pathological accumulation of calcium phosphate minerals in macrophages (27). To figure out the mechanistic distinction between Col’s effects on crystal accumulation and its potential direct regulation of NLRP3 Ser 5 phosphorylation, we treated human VSMCs with Col in the presence or absence of hydroxyapatite, the predominant calcium phosphate mineral that accumulates in the vascular wall and drives vascular calcification (28). Our results showed that Col reduced NLRP3 phosphorylation at Ser 5, as well as the levels of downstream targets (mature form of IL-1β), under both conditions (Supplementary material online, Fig. S4, *A* and *B*). Importantly, this effect occurred at an early stage of the calcification process. Alizarin Red S staining indicated that neither Col nor hydroxyapatite significantly altered calcification during short-term treatment. However, long-term treatment with Col reduced calcium accumulation regardless of hydroxyapatite presence (Supplementary material online, Fig. S4, *C*–*E*). Together, these results suggest that Col inhibits NLRP3 activation *via* a crystal-independent mechanism, most likely through direct regulation of Ser 5 phosphorylation.

Furthermore, we aim to investigate the mechanism by which Col regulates the phosphorylation of NLRP3. Protein phosphatase 2Ac (PP2Ac) is a phosphatase and has been demonstrated to play a crucial role in dephosphorylation and activation of NLRP3 (23, 25, 26). To explore whether PP2Ac is involved in the regulation of Col on NLRP3 activation, we detected PP2Ac expression and found that PP2Ac expression was enhanced by high phosphate, while Col had little effects on PP2Ac (Supplementary material online, Fig. S5). To explore the direct role between PP2Ac and NLRP3 activity, we transfected cells with PP2Ac siRNA and found that PP2Ac inhibition largely reduced IL-1β and caspase-1 expression and ASC oligomerization. In addition, Col had little reduction effects on expression of IL-1β and caspase-1, as well as ASC oligomerization in PP2Ac knockdown cells (Fig. 4, *F*–*H*). More importantly, we indicated that phosphorylated NLRP3 was significantly enhanced in PP2Ac knockdown cells (Fig. 4*I*). Additionally, our results showed that secretion of IL-1β was reduced by PP2Ac inhibition (Fig. 4*J*). Taken together, we suggested that PP2Ac is involved in Col-regulated calcification.

### Col enhances the activity of sirtuin 2 to reduce the acetylation and activity of PP2Ac

**Figure 5:**
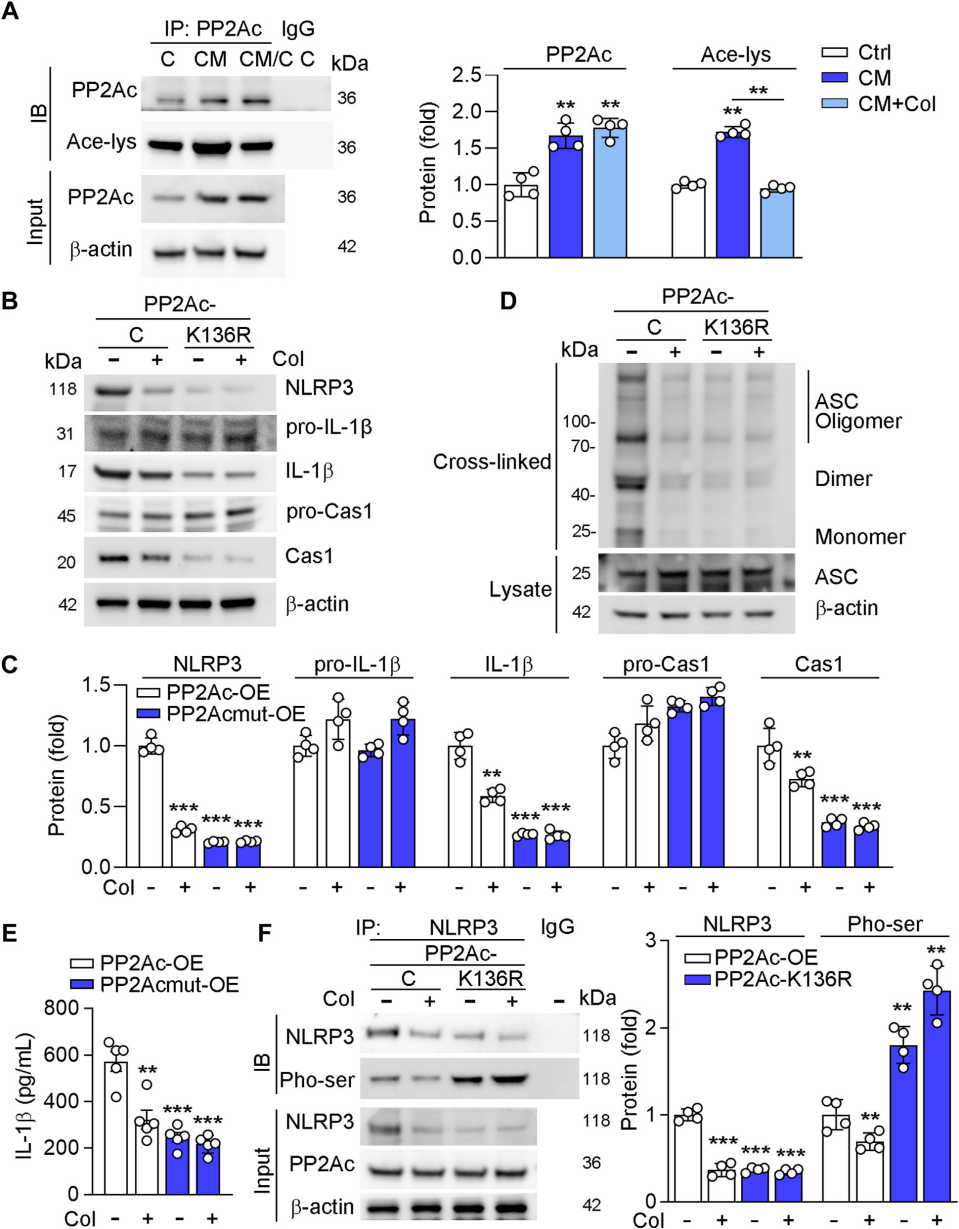
**Col regulated NLRP3 activity is related to the deacetylation of PP2Ac.***A*, Human VSMCs were cultured in control medium or CM or CM containing col for 4 days. *B*–*F*, human VSMCs were transfected with PP2Ac or PP2Ac-K136R, then treated with Col for another 4 days. For co-IP assay, cell lysates were immunoprecipitated with control IgG antibody or anti-PP2Ac antibody or anti-NLRP3 antibody, followed by determination protein expression by Western blot using the indicated antibodies (*A* and *F*, n = 4). For determination protein expression, cell lysates were used for conduction of Western blot with quantification of band density (*B* and *C*, n = 4). For determination ASC oligomerization, cell lysates were used for extracted cross-linked cytosolic pellets and determined by Western blot (*D*, n = 4). Cell medium was collected for determination of IL-1β levels using the Elisa assay kit (*E*, n = 5). For all panels, data are presented as mean ± SD. ∗∗*p* < 0.01, ∗∗∗*p* < 0.001 by one-way ANOVA with Bonferroni correction test. ASC, apoptosis-associated speck-like protein with a caspase recruitment domain; CM, calcification medium; Col, colchicine; NLRP3, NLR family pyrin domain-containing 3; PP2Ac, protein phosphatase 2Ac; VSMCs, vascular smooth muscle cells.

In this study, we found that Col had little effects on PP2Ac expression, while PP2Ac inhibition can regulate NLRP3 activity. We then supposed that Col might regulate the activity of PP2Ac. It has been reported that activation of PP2Ac can be regulated by its acetylation (29). We found high phosphate treatment enhanced acetylation of PP2Ac, indicating enhancing the activity. In contrast, Col reduced calcification medium (CM)-induced PP2Ac acetylation (Fig. 5*A*), indicating the activity of PP2Ac may be regulated by the Col. Deacetylated PP2Ac at Lys 136 resulted in PP2Ac inactivation (29). Therefore, we constructed a plasmid for mutation of Lys at 136 to arginine (Arg/R, the nonacetylated state of the lysine residue), and named as PP2Ac-K136R. As shown in Figure 5, *B*–*E*, our results showed that deacetylated PP2Ac largely reduced expression of IL-1β and Caspase-1, oligomerization of ASC, as well as IL-1β secretion. More importantly, we showed that phosphorylation of NLRP3 was increased in PP2Ac-K136R transfected cells (Fig. 5*F*). Therefore, these results suggested that the activation of NLRP3 was regulated by the deacetylation and activation of PP2Ac.

**Figure 6:**
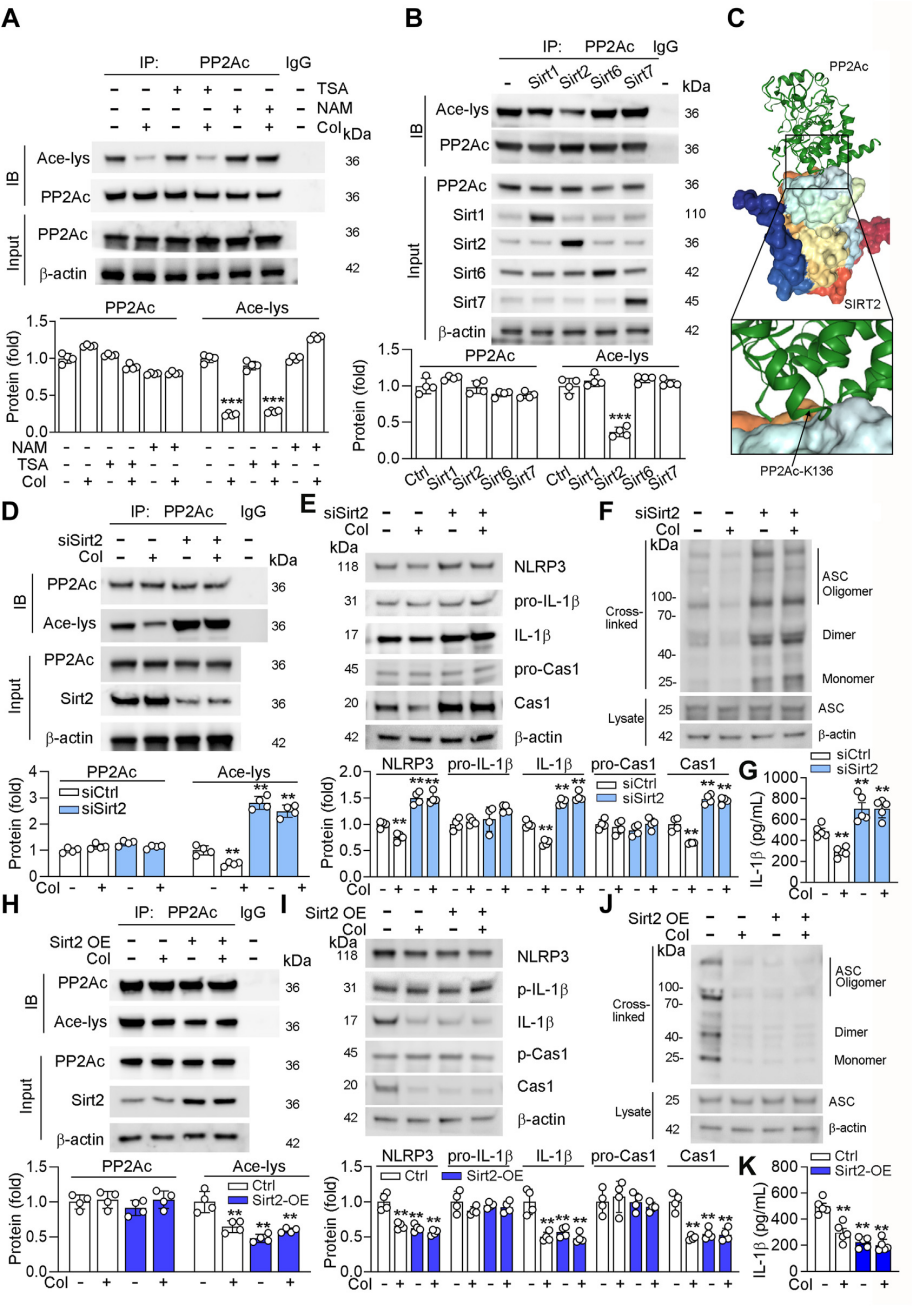
**The activity of PP2Ac is regulated by Sirt2**. *A*, human VSMCs were treated with TSA (2 ng/ml), NAM (5 mM), or Col (5 nM) for 24 h. *B*, *D*–*K*, human VSMCs were transfected with indicated overexpression adenovirus or SIRT2 siRNA or SIRT2 overexpression adenovirus for 48 h, then received Col (5 nM) treatment for 24 h. For co-IP assay, cell lysates were immunoprecipitated with control IgG antibody or anti-NLRP3 or anti-acetylated lysine or anti-PP2Ac antibody, followed by determination protein expression by Western blot using the indicated antibodies (*A*, *B*, *D* and *H*, n = 4). For determination protein expression, cell lysates were used for conduction of Western blot with quantification of band density (*E* and *I*, n = 4). For determination ASC oligomerization, cell lysates were used for extracted cross-linked cytosolic pellets and determined by Western blot (*F* and *J*, n = 4). Cell medium was collected for determination of IL-1β levels using the Elisa assay kit (*G* and *K*, n = 5). *C*, SIRT2-PP2Ac docking with the HDOCK server. *High* magnification of *boxed areas* is presented on the *right*. *Arrow* indicates PP2Ac protein K136 site. For all *panels*, data are presented as mean ± SD. ∗∗*p* < 0.01, ∗∗∗*p* < 0.001 by one-way ANOVA with Bonferroni correction test. ASC, apoptosis-associated speck-like protein with a caspase recruitment domain; Col, colchicine; NLRP3, NLR family pyrin domain-containing 3; PP2Ac, protein phosphatase 2Ac; sirt2, sirtuin 2; VSMCs, vascular smooth muscle cells.

Histone deacetylases and sirtuins (SIRTs) are well known deacetylases. To figure out which deacetylase regulates the activity of PP2Ac, we treated cells with trichostatin A (an inhibitor of histone deacetylase) and niacinamide (an inhibitor of SIRTs). As shown in Figure 6*A*, we found that trichostatin A had little effects on of PP2Ac acetylation, while niacinamide blocked the Col-reduced PP2Ac acetylation, indicating SIRTs are involved in regulation of PP2Ac deacetylation. Then, we transfected cells with SIRTs to explore which one is involved in the acetylation regulation of PPA2c. As shown in Figure 6*B*, we found that only overexpression of Sirt2 reduced acetylation of PPA2c. HDOCK server (http://hdock.phys.hust.edu.cn/), a highly integrated suite of protein–protein docking information, predicted the docking information about SIRT2-PP2Ac, helping to elucidate SIRT2 and PP2Ac-Lys136 interactions (Fig. 6*C*). Furthermore, we transfected cells with Sirt2 siRNA or overexpression adenovirus and treated cells with Col. Our results showed that acetylation of PP2Ac was enhanced by Sirt2 knockdown, while reduced by Sirt2 overexpression (Fig. 6, *D* and *H*). Moreover, Col reduced acetylation of PP2Ac in control cells but had little effects on Sirt2 knockdown or overexpression cells, indicating that Col regulates PP2Ac acetylation in a Sirt2-dependent manner (Fig. 6, *D* and *H*). Furthermore, we also showed that NLPR3 inflammasome activation was enhanced in Sirt2 knockdown (Fig. 6, *E*–*G*) but reduced in Sirt2-overexpressed cells (Fig. 6, *I*–*K*). In addition, we also showed that Col had little role in both Sirt2 knockdown and overexpression cells. Taken together, the above results showed that Sirt2 expression is involved in Col-reduced activation of NLRP3.

### Col-attenuated calcification is dependent on Sirt2 expression

**Figure 7:**
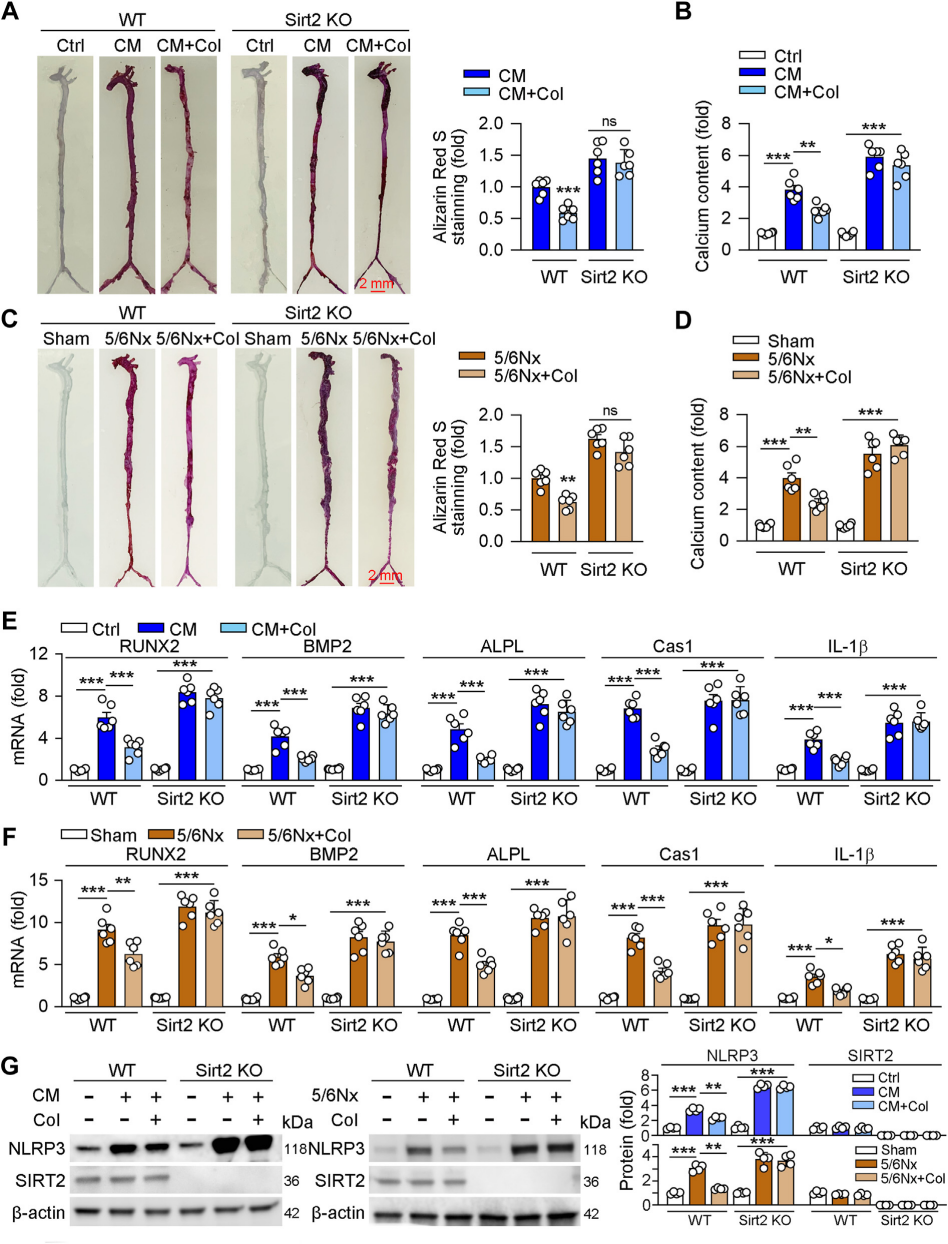
**The protective role of Col on calcification is dependent on Sirt2**. C57BL/6J or Sirt2 knockout (Sirt2 KO) mice used VD3 plus nicotine or 5/6 nephrectomy (5/6Nx) combined with high phosphate diet to construct vascular calcification mouse model*. A* and *C*, vascular calcification in whole aorta (*A* and *C*) was determined by Alizarin Red S staining followed by quantitative analysis (*right panels* of *A* and *C*, n = 6). *B* and *D*, calcium content was were determined by indicated assay kit (*B* and *D*, n = 6). *E*–*G*, mRNA or protein expression was determined by or qRT-PCR (*E* and *F*, n = 6) Western blot with quantification of band density (*G*, n = 4). For all *panels*, data are presented as mean ± SD. ∗*p* < 0.05, ∗∗*p* < 0.01, ∗∗∗*p* < 0.001 by one-way ANOVA with Bonferroni correction test. Col, colchicine; sirt2, sirtuin 2; VD3, Vitamin D3.

To further determinate the role of Sirt2 on Col-reduced vascular calcification. We used Sirt2 knockout (Sirt2 KO) and wildtype mice for constructing vascular calcification mouse models using the VD_3_ plus nicotine and 5/6 nephrectomy plus nephrectomy along with high phosphate diet. As shown in Figure 7, *A* and *C*, we found that calcification in Sirt2 KO mouse aorta was sever than those in WT mice in both VD3 plus nicotine and 5/6 nephrectomy mouse models. More importantly, Col had little protective role in vascular calcification in Sirt2 KO mice (Fig. 7, *A* and *C*). Consistently, the results of calcium content and protein expression of calcification-related markers were similar to the results of Alizarin red S staining (Fig. 7, *B*, *D*, *E* and *F*). Moreover, we also found that Cas1, IL-1β, as well as NLRP3 were in a higher level in Sirt2 KO mouse than those in WT mouse. Additional, Col had little reduction role on NLRP3, Cas1, and IL-1β in Sirt2 KO mouse (Fig. 7, *E*–*G*). The above results indicate that Col-attenuated vascular calcification depends on the expression of Sirt2.

## Discussion

In this study, we demonstrated the essential role of Col on vascular calcification inhibition. Notably, the protective effect of Col against vascular calcification in mice is dependent on Sirt2 expression. Our study revealed that activation of Sirt2 can inhibit NLRP3 activity by regulating its phosphorylation through PPA2c. Furthermore, we indicated that the activation of PPA2c was regulated by Sirt2 though its deacetylation of PP2Ac. Therefore, our data indicated that Col reduces vascular calcification though the Sirt2-PP2Ac-NLRP3 pathway. Our findings not only highlight the importance of Col in protecting against vascular calcification but also uncover the novel mechanism by which Col regulates NLRP3.

More and more studies have shown that members of SIRT family, such as Sirt1, Sirt3, and Sirt6, play fundamental role in vascular calcification. Studies have shown that the downregulation of Sirt1 due to aging is associated with abnormal phenotypes of smooth muscle cells, vascular calcification, and cardiovascular events in diabetic patients (30, 31). The expression of Sirt3 is negatively correlated with calcification progression (32). Researchers have reported that both Sirt3 and Sirt6 are involved in vascular calcification caused by chronic kidney disease and aging (33, 34, 35). Sirt2 is a well-known histone deacetylase, one of the members of the SIRT family, which mediated multiple biological processes. Researches have shown that Sirt2 plays important roles in cardiovascular diseases. Sirt2 protects mice against both aging-related and Ang II-induced cardiac hypertrophy through activating AMPK by deacetylating LKB1, indicating that Sirt2 may be a potential target for the treatment of cardiac hypertrophy (36). In addition to regulating AMPK pathway, Sirt2 can also deacetylate NFATc2 and inhibit the pathological of myocardial hypertrophy (37). In myocardial ischemia-reperfusion injury, one study showed that Sirt2 performs the protective role, but another one indicated that Sirt2 can aggravate the damage (38, 39), and the reason for the opposite role needs to be further investigation. Our previous study indicated that Sirt2 can reduce atherosclerosis by regulating the phenotype of macrophages (40). Moreover, Chen *et al.* reported that Sirt2 can not only protect cardiac hypertrophy but also attenuate aging-induced vascular remodeling (36, 41). However, there are few studies about the role of Sirt2 on vascular calcification. In the present study, we showed that knockout of Sirt2 aggravated the process of vascular calcification which was induced by the VD_3_ plus nicotine and 5/6 nephrectomy combined with high phosphate diet (Fig. 7).

NLRP3 inflammasome activation contributes to a variety type of diseases, which is involved in the activation of inflammation process. In has been proved that Col can target NLRP3 for amelioration abdominal aortic aneurysms, atherosclerosis-associated inflammation, and viral myocarditis in mice (42, 43). More importantly, Col is widely used in clinic. The randomized trials demonstrated that the risk of cardiovascular events was significantly lower among those who received Col treatment at the dose of 0.5 mg/day (16, 19, 44). However, the role of Col on vascular calcification is still unclear, as well as the detailed molecular regulation on NLRP3. In the current study, we found that Col can inhibit high phosphate-induced calcium deposition in VSMC and VD_3_ plus nicotine-induced vascular calcification in mice (Figs. 1 and 2). In addition, the dosage of Col used in the study is in accordance with that in clinic. Previous study indicated that the activation of NLRP3 can regulate the posttranslational modification, including phosphorylation and acetylation (23, 24, 25, 26). In this study, we demonstrated that Col-reduced NLRP3 activation was not dependent on the acetylation but mediated by enhancing its phosphorylation at Ser 5 (Fig. 4). However, further *in vivo* studies are needed to demonstrate the role of NLRP3 phosphorylation at Ser 5 in vascular calcification. Previous study has shown that Col reduces pathological accumulation of calcium phosphate minerals in macrophages (27). Our findings indicate that Col inhibits NLRP3 activation independently of crystal accumulation in VSMCs, primarily through suppressing Ser 5 phosphorylation (Fig. S4). On the other hand, the protective role of Col *in vivo* may also be attributed to its ability to reduce calcium deposition in macrophages. The potential crosstalk between macrophages and VSMCs, possibly mediated through inflammatory signaling or extracellular vesicles, warrants further investigation.

Previous study has shown that IL-1β is a key regulator of NLRP3 itself (45). Moreover, it has been demonstrated that AKT phosphorylates NLRP3 at Ser 5, preventing oligomerization while simultaneously stabilizing NLRP3 by reducing its ubiquitination on K496, thereby inhibiting proteasomal degradation (46). Given this mechanism, it is likely that Ser 5 dephosphorylation not only promotes oligomerization but also enhances NLRP3 degradation, which could contribute to the observed activation dynamics in our study. The other study has shown that AKT was activated by CM (47). Our results showed that both NLRP3 expression and phosphorylation can be regulated by Col, which indicated that other pathways, such as feedback regulation mechanism and AKT pathway may also attend the calcification process. However, it remains possible that additional modifications, such as ubiquitination, SUMOylation, or other phosphorylation events, can also contribute to NLRP3 activation following Col treatment.

PP2Ac is a phosphatase, involved in dephosphorylation and activation of NLRP3 (23, 25, 26). Previous study has shown that the activity of PP2Ac was mediated by the acetylation, which was regulated by histone deacetylase 5 (29). However, in this study, we indicated that Col-affected PP2Ac deacetylation at Lys 136 is dependent on Sirt2 (Fig. 6). It has shown that histone deacetylase 5 deacetylates the PP2A for positively regulating nuclear factor kappa *B* (NF-κB) signaling (29). Whereas, our study found that BAY11 to 7082 (an inhibitor of NF-κB) had little effects on mature IL-1β and cleaved caspase-1 in both PPA2c knockdown or overexpressed cells (Supplementary material online, Fig. S6), indicating that NF-κB is not involved in the process of Col-regulated vascular calcification. Therefore, we demonstrated that Col-inhibited activation of NLRP3 is dependent on the regulation of Sirt2-PP2Ac pathway.

Taken together, we suggested that Col could be efficiency used for the treatment of vascular calcification. In mechanism, Col activates Sirt2, which deacetylates PP2Ac, thereby inhibiting NLRP3 activation by enhancing its phosphorylation. Therefore, our study not only suggests the potential therapeutic use of Col for vascular calcification but also explores the mechanism by which Col inhibits NLRP3. More importantly, we also identify that Sirt2 may be a novel target for the treatment of vascular calcification.

## Experimental procedures

### Reagents

L-nicotine was purchased from GLPBIO. VD_3_ and Alizarin Red S solution were purchased from Solarbio. Transfection reagent was purchased from GLPBIO. PP2Ac and Sirt2 and control siRNA were purchased from Santa Cruz Biotechnology. Calcium assay kit was purchased from Beyotime. IL-1β Elisa assay kit was purchased from Nanjing Jiancheng Bioengineering Institute. Antibodies used in this study were listed in Table 1. Other reagents were purchased from Millipore Sigma.

**Table 1:** Antibodies used in this study

Antibodies	Source	Identifier
BMP2 (1:1000)	Abcam	Cat# ab214821; RRID:AB_2814695
ALPL (1:1000)	Novus	Cat# AF2910; RRID:AB_664062
RUNX2 (1:1000)	Abcam	Cat# ab192256; RRID:AB_2713945
NLRP3 (1:1000)	Abcam	Cat# ab263899; RRID:AB_2889890
IL-1β (1:1000)	Cell Signaling Technology	Cat# 31202; RRID:AB_2799001
Cleaved-IL-1β (1:1000)	Cell Signaling Technology	Cat# 52718; RRID:AB_2799421
Caspase-1 (1:1000)	Cell Signaling Technology	Cat# 3866; RRID:AB_2069051
Cleaved Caspase-1 (1:1000)	Cell Signaling Technology	Cat# 4199; RRID:AB_1903916
ASC (1:1000)	Cell Signaling Technology	Cat# 67824; RRID:AB_2799736
SMA (1:1000)	Abcam	Cat#ab7817; RRID:AB_262054
CD68 (1:1000)	Abcam	Cat# ab283654; RRID:AB_2922954
OPN (1:1000)	Proteintech	Cat# 22952-1-AP; RRID:AB_2783651
Ace-lys (1:1000)	Abcam	Cat# ab21623; RRID:AB_446436
Pho-ser (1:1000)	Abcam	Cat# abab17464; RRID:AB_443891
PP2Ac (1:1000)	Proteintech	Cat# 13482-1-AP; RRID:AB_2169485
Sirt1 (1:1000)	Novus	Cat# NBP2-27205
Sirt2 (1:1000)	Abcam	Cat# ab211033; RRID:AB_2927614
Sirt6 (1:1000)	Abcam	Cat# ab191385
Sirt7 (1:1000)	Proteintech	Cat# 12994-1-AP; RRID:AB_10644276
β-actin (1:1000)	Abcam	Cat# ab8226; RRID:AB_306371

### Cell culture

Human VSMC cells were purchased from Pricella (Wuhan) and cultured in Dulbecco's modified Eagle's medium supplemented with 10% fetal bovine serum, 2 mmol/L glutamine, and 50 μg/ml penicillin/streptomycin.

### Animals and *in vivo* experiments

Male C57BL/6J mice were purchased from GemPharmatech (Nanjing), and Sirt2 KO mice were purchased from Cyagen Biosciences (Guangzhou) and housed at the Animal Center of Hefei University of Technology. The protocol of animal studies (#HFUT20220506003) was approved by the Ethics Committee of Hefei University of Technology and conformed to the Guide for the Care and Use of Laboratory Animals published by the NIH (NIH publication no.:85–23, revised 1996).

Vascular calcification was induced by VD_3_ plus nicotine or 5/6 nephrectomy combined with high phosphate diet as described previously (12, 13). Briefly, for the VD_3_ plus nicotine model, mice received intragastric administration of nicotine (25 mg/kg) at 0 h and 12 h on the first day and subcutaneous injection of VD_3_ (5.5 × 10^5^ IU/kg/day) for 3 days. For the 5/6 nephrectomy combined with high phosphate diet model, two-thirds of the left kidney was removed from mice in model group in the first week, and the right kidney was removed in the second week, and after 1 week of recovery, a diet containing 1.8% phosphate was fed. The mice in control group underwent sham surgery and fed a diet containing 0.5% phosphate. Then, mice received intragastric administration of Col (0.05 mg/kg/day) or vehicle for 3 weeks (for the VD_3_ plus nicotine mouse model) or 8 weeks (for the 5/6 nephrectomy mouse model). At the end of experiments, mice were anesthetized in a CO_2_ chamber, followed by collection of aorta and blood samples.

### *In vitro* or *ex vivo* calcification induction

CM was prepared as described previously (13), containing 3 mmol/L inorganic phosphate (Na_2_HPO_4_/NaH_2_PO_4_, 1:2) in Dulbecco's modified Eagle's medium with 2% FBS. Human VSMCs were incubated in CM for 3 to 7 days, and the medium was replaced every other day. For the *ex vivo* experiment, thoracic aorta was collected from male C57BL/6J mice (8-week-old) and cultured in CM for 7 days.

### Alizarin Red S and immunofluorescence staining

To determine calcification deposition *in vitro* or *ex vitro* or *in vivo*, Alizarin Red S staining was conducted, followed by quantification the absorbance of the extracted dye as described previously (13). Protein expression of ALPL, BMP2, RUNX2, and NLRP3 was determined using immunofluorescence staining by the indicated antibodies (13). Images were obtained by a Zeiss microscope (Oberkochen) and qualitied the mean fluorescence intensity using Image J software (NIH).

### Western blot and qRT-PCR

Total proteins extracted from mouse aorta or cells were used to determine the expression of ALPL, ASC, BMP2, RUNX2, CD68, SMA, NLRP3, pro-IL-1β, IL-1β, pro-caspase-1, caspase-1, PP2Ac, Sirt2, and GAPDH by Western Blot. Total proteins extracted from cells were used for conduction of IP assay with indicated antibodies, then determined the expression of related acetylation or phosphorylation protein by Western blot. The signals were detected by Chemiscope 3000 mini (Qin Xiang), and the band density was quantified using Photoshop software.

Total RNA was extracted from cells using Trizol. cDNA was synthesized, and qRT-PCR was performed using the AceQ SYBR qPCR Master Mix reverse transcriptase kit using LightCycler96 (Vazyme) with indicated primers as listed in Table 2. mRNA expression was normalized by GAPDH mRNA in the corresponding samples.

**Table 2:** Primers used in qPCR

Gene	Forward primer (5′→3′)	Reverse primer (5′→3′)
m*Runx2* (ID: 12,393)	*CCAACCGAGTCATTTAAGGCT*	*GCTCACGTCGCTCATCTTG*
m*Bmp2* (ID: 12,156)	*GGGACCCGCTGTCTTCTAGT*	*TCAACTCAAATTCGCTGAGGAC*
m*Alpl* (ID: 11,647)	*CCAACTCTTTTGTGCCAGAGA*	*GGCTACATTGGTGTTGAGCTTTT*
m*Cas1* (ID: 12,362)	*ACAAGGCACGGGACCTATG*	*TCCCAGTCAGTCCTGGAAATG*
m*Il-1β* (ID: 16,176)	*GCAACTGTTCCTGAACTCAACT*	*ATCTTTTGGGGTCCGTCAACT*
m*β-actin* (ID:11,461)	*GGCTGTATTCCCCTCCATCG*	*CCAGTTGGTAACAATGCCATGT*
h*α-SMA* (ID: 59)	*GCGTGGCTATTCCTTCGTTA*	*ATGAAGGATGGCTGGAACAG*
h*CD68* (ID: 968)	*TGGGGCAGAGCTTCAGTTG*	*TGGGGCAGGAGAAACTTTGC*
h*OPN* (ID: 6696)	*GAAGTTTCGCAGACCTGACAT*	*GTATGCACCATTCAACTCCTCG*
h*GAPDH* (ID: 2597)	*GGAGCGAGATCCCTCCAAAAT*	*GGCTGTTGTCATACTTCTCATGG*

### ASC oligomerization assay

The ASC oligomerization assay was performed according to the previous study (48). In brief, after treatment, cells were lysed in the lysis buffer (50 mM Tris-HCl pH 7.4, 150 mM NaCl, and 0.5% Triton X-100), which containing the EDTA-free protease inhibitor and phosphatase inhibitor. The lysates were centrifuged at 6000*g* for 15 min at 4 °C, and the resulting supernatants and pellets were designated as Triton-soluble and Triton-insoluble fractions, respectively. To detect ASC oligomerization, the Triton-insoluble pellets were washed twice with TBS buffer and then resuspended. The resuspended pellets were crosslinked with disuccinimidyl suberate (2 mM) at 37 °C for 30 min and followed by centrifugation at 6000*g* for 15 min. The pellets were then dissolved in SDS sample buffer.

### Data analysis

The data were obtained from at least three independent experiments and expressed as mean ± SEM. All data were statistically analyzed by GraphPad Prism 8.0 software, and the statistical analysis was performed by one-way ANOVA with Bonferroni correction test. Significant difference was considered when *p* < 0.05.

## Data availability

All data presented are contained within the main manuscript and supporting information.

## Supporting information

This article contains supporting information.

## Conflict of interest

The authors declare that they have no conflicts of interest with the contents of this article.
