# Supplementary material for: Colchicine inhibits vascular calcification by suppressing inflammasome activation through the enhancement of the Sirt2-PP2Ac signaling pathway
Source: J Biol Chem. 2025 Jun 14;301(7):110381. doi: 10.1016/j.jbc.2025.110381 (PMC12274824; doi:10.1016/j.jbc.2025.110381)
Supplement: Supplementary material [file mmc1.docx]

**Colchicine inhibits vascular calcification by suppression inflammasome activation through the enhancement of the Sirt2-PP2Ac signaling pathway**

Shu Yang^1,#^, Heming Huang^1,#^, Kewei Jiang^1,#^, Ying Peng^2^, Zhen Liang^1^, Xinyu Gong^2^, Lixing Li^1^, Yanchun Li^1^, Buchun Zhang^3,^*, Yuanli Chen^2,^*, Xiaoxiao Yang^2,4,^*

^1^Department of Geriatrics, The First Affiliated Hospital of Southern University of Science and Technology, Shenzhen, Guangdong 518020, China; ^2^Anhui Provincial International Science and Technology Cooperation Base for Major Metabolic Diseases and Nutritional Interventions, College of Food and Biological Engineering, Hefei University of Technology, Hefei, Anhui, 230601, China; ^3^Department of Cardiology, the First Affiliated Hospital of USTC, Division of Life Sciences and Medicine, University of Science and Technology of China, Hefei, Anhui, 230001, China

^4^Lead contact

^#^These authors contributed equally

Supplemental Figures and Figure legends


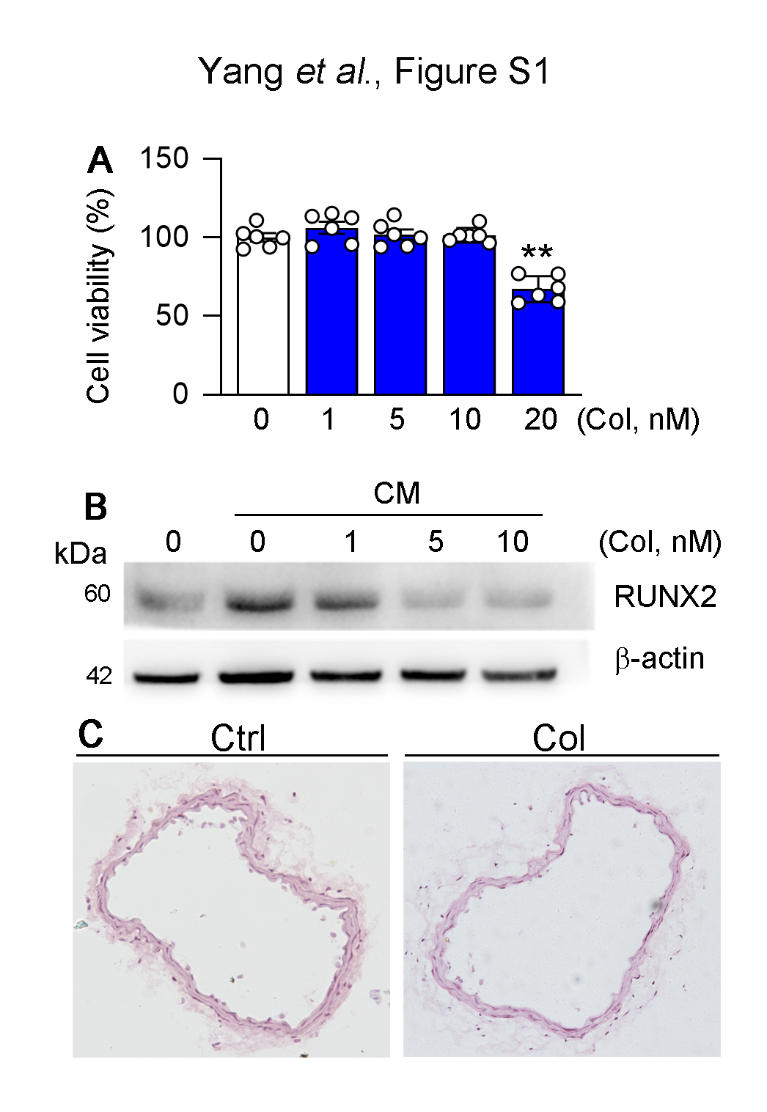


**Figure S1 Low concentration of Col has little toxic effects *in vitro* and *ex-vitro***

**A.** Human VSMCs were cultured in 2% FBS and 1% P/S medium and treated with indicated concentration of Col for 2 days. MTT assay was conducted for determination of cell viability (n = 6). **B**. Human VSMCs were cultured in 2% FBS and 1% P/S medium (control medium) or high phosphate medium (CM) or CM contain Col for 4 days. RUNX2 protein expression was determined by Western blot. **C**. Thoracic aortas were collected from C57BL/6J mice and cut into 5 mm long aortic rings, then cultured in 2% FBS and 1% P/S medium or medium contain Col (5 nM) for 14 days. HE staining was used to determine cellular morphology. Data are presented as mean ± SD. **P < 0.01 by one-way ANOVA with Bonferroni correction test.


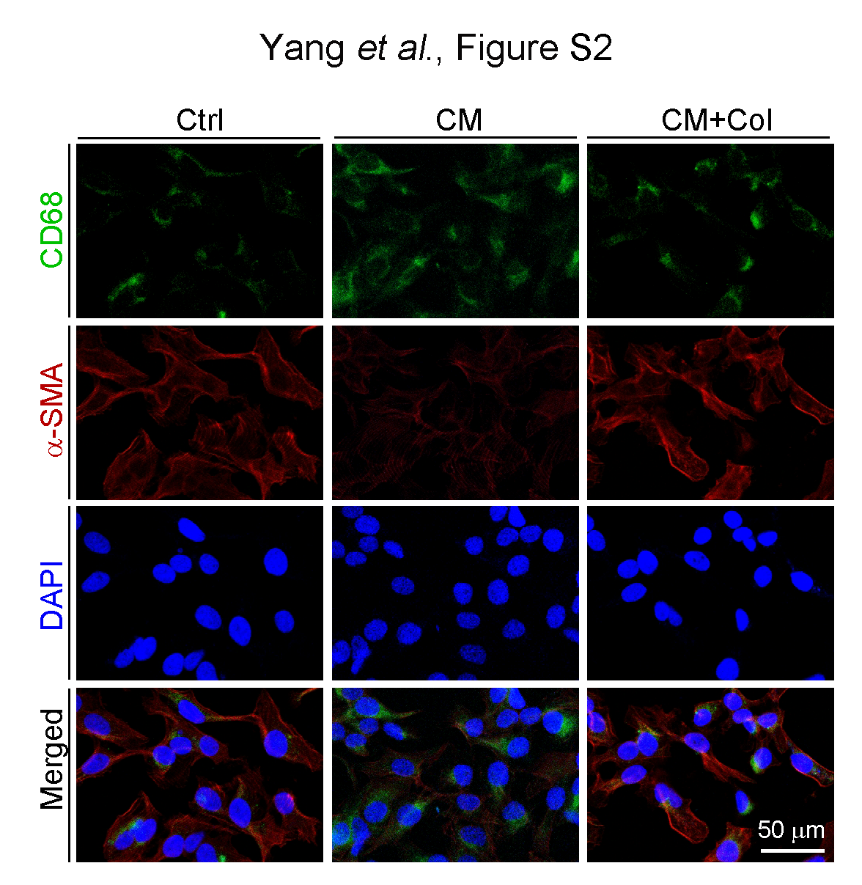


**Figure S2 Col regulates VSCM phenotype transformation.**

Human VSMCs were cultured in 2% FBS and 1% P/S medium or high phosphate medium (CM) or CM contain Col (5 nM) for 4 days. CD68 and SMA expression was determined by immunofluorescent staining.


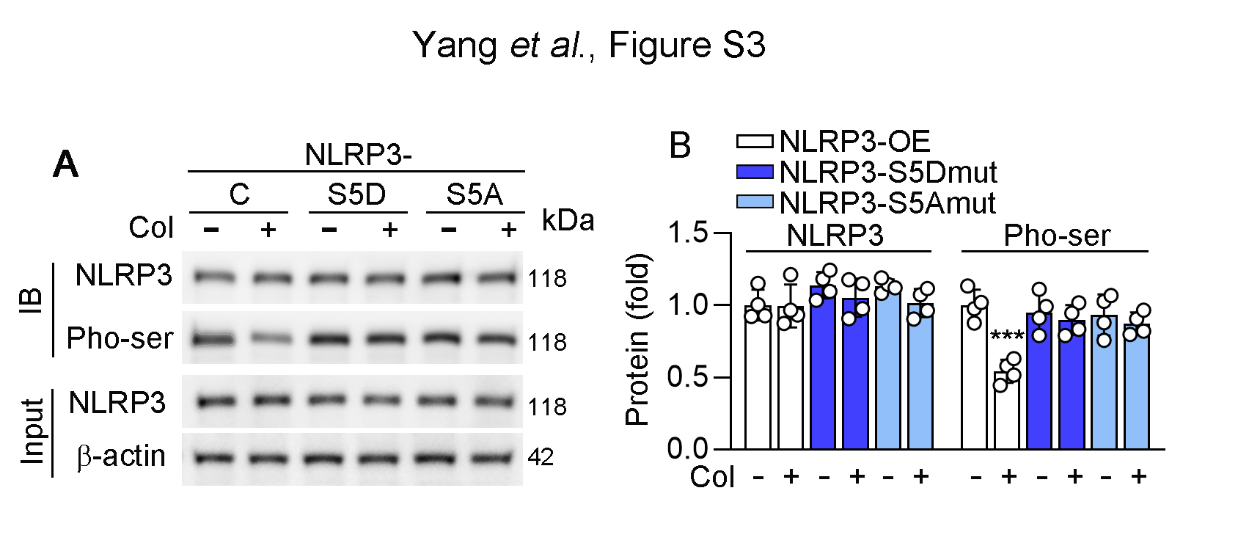


**Figure S3 Col has little effects on NLRP3 phosphorylation in mutated NLRP3 transfected cells.**

Human VSMCs were transfected with NLRP3, NLRP3-S5D, NLRP3-S5A, and treated with Col for 4 days. For co-IP assay of NLRP3, cell lysates were immunoprecipitated with control IgG antibody, or anti-NLRP3 antibody, followed by determination protein expression by Western blot using the indicated antibodies. For all panels, data are presented as mean ± SD. ***P < 0.001 by one-way ANOVA with Bonferroni correction test.


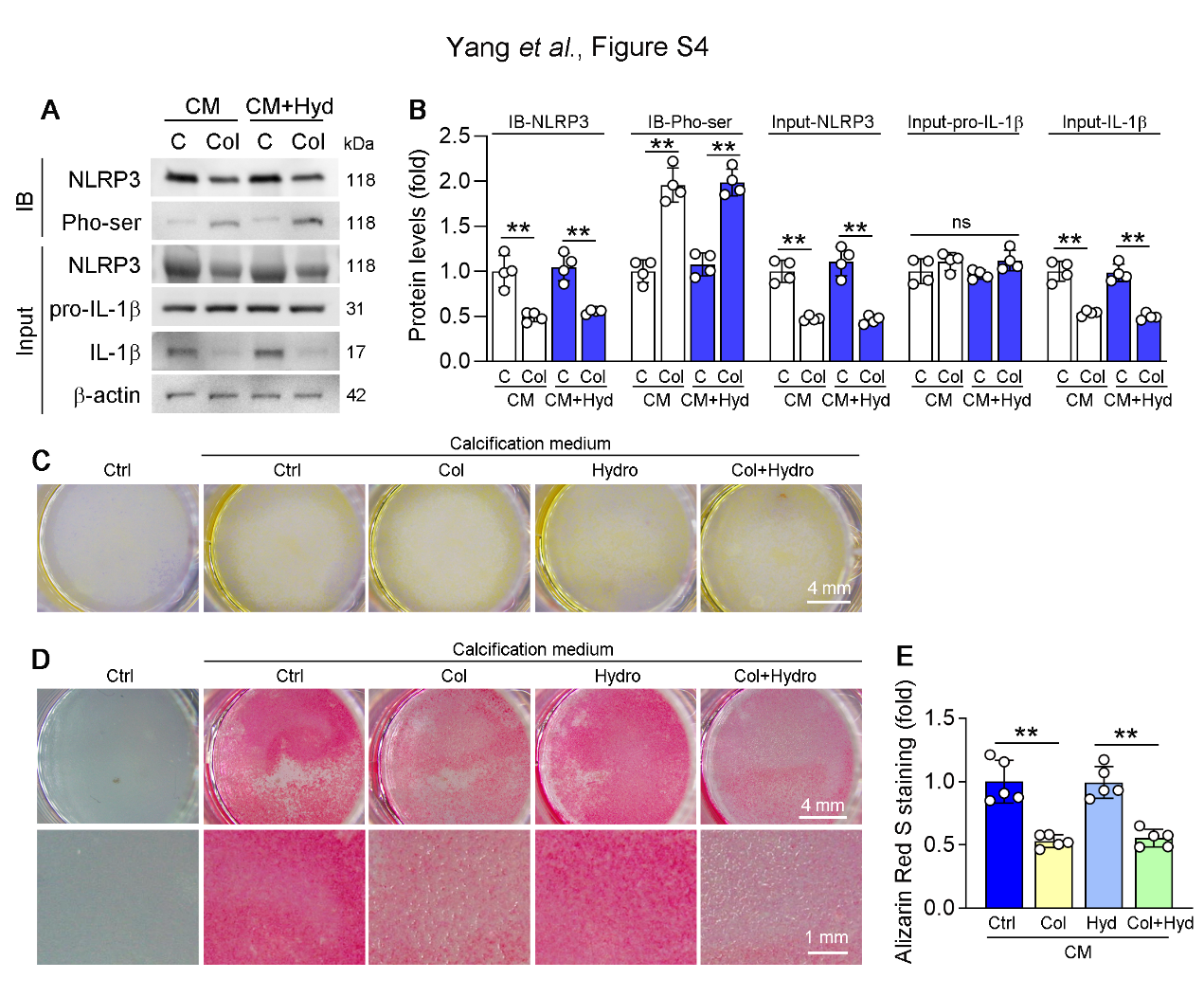


**Figure S4 Col inhibits NLRP3 activation via a crystal-independent mechanism.**

Human VSMCs were treated with Col (5 nM) in the in the presence or absence of hydroxyapatite (Hyd or Hy, 10 μg/mL) for 24 h (A-C) or 4 days (D and E). A and B: For co-IP assay of NLRP3, cell lysates were immunoprecipitated with control IgG antibody, or anti-NLRP3 antibody, followed by determination protein expression by Western blot using the indicated antibodies (n = 4). C-E: Alizarin red S staining was used to determine calcification, followed by quantitative analysis (n = 5). For all panels, data are presented as mean ± SD. **P < 0.001 by one-way ANOVA with Bonferroni correction test.


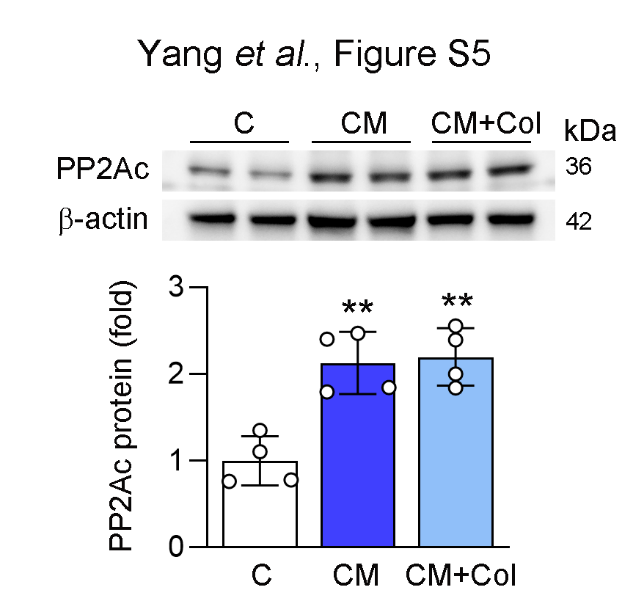


**Figure S5 Col has little effects on PP2Ac expression**

Human VSMCs were cultured in 2% FBS and 1% P/S medium (control medium) or high phosphate medium (CM) or CM contain Col (5 nM) for 4 days. PP2Ac protein expression was determined by Western blot with quantification of band density (n = 4). For all panels, data are presented as mean ± SD. **P < 0.01 by one-way ANOVA with Bonferroni correction test.


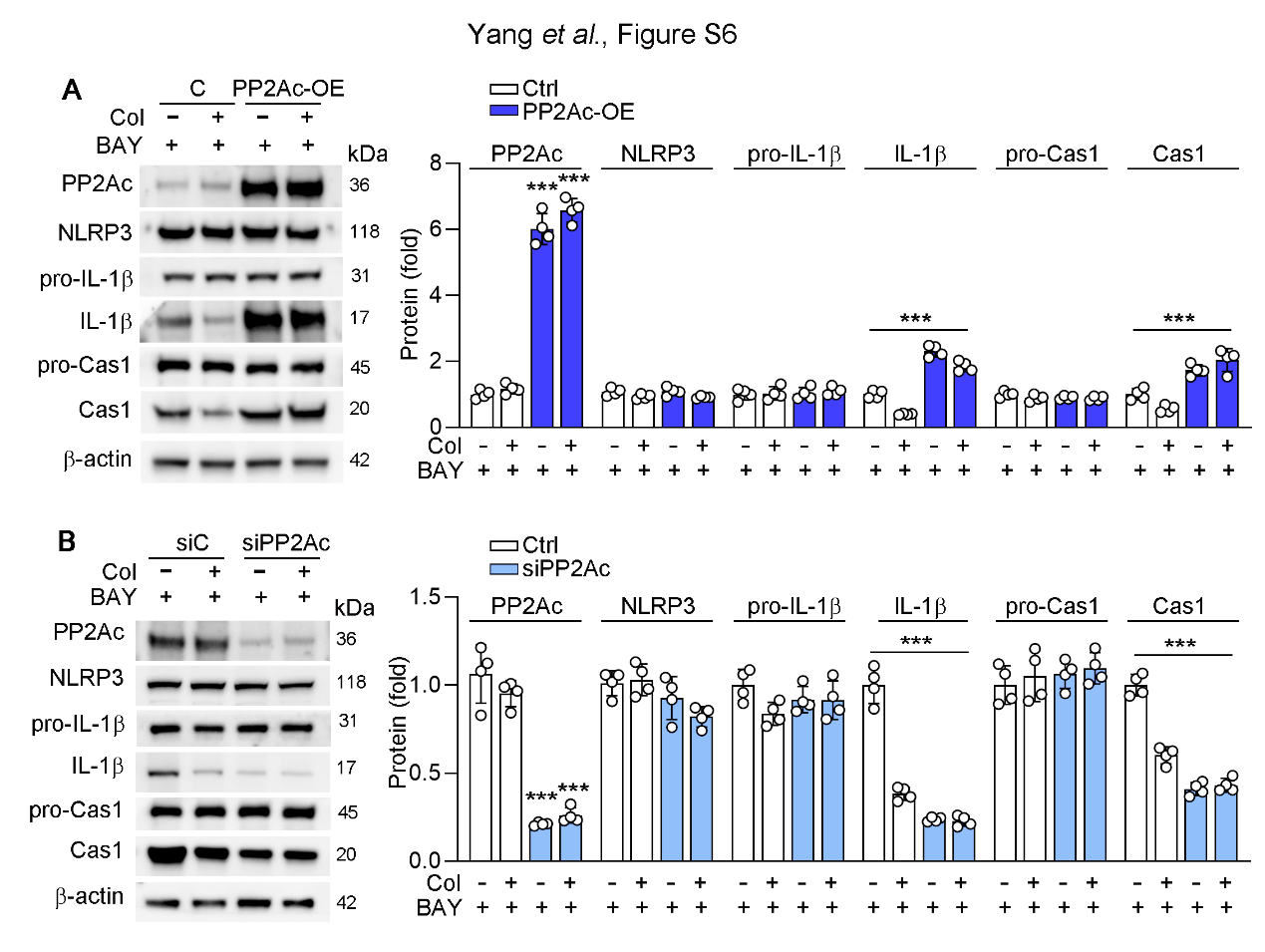


**Figure S6 NF-κB pathway is not involved in Col-regulated vascular calcification**

Human VSMCs were transfected with PP2Ac overexpression adenovirus or siRNA for 48 h, then received Col (5 nM) and BAY (10 μM) treatment for 24 h. PP2Ac, NLRP3, pro-IL-1β, IL-1β, pro-caspase 1 and caspase 1 protein expression was determined by Western blot with quantification of band density (n = 4). For all panels, data are presented as mean ± SD. ***P < 0.001 by one-way ANOVA with Bonferroni correction test.
